# Thrips and plant viruses: a comprehensive virome analysis in tropical agriculture

**DOI:** 10.3389/fmicb.2025.1540883

**Published:** 2025-04-28

**Authors:** Qikai Zhang, Chunxi Cheng, Baoqian Lyu, Yulin Gao, Lilin Chen, Liang Peng, Zhengfu Yue, Hui Lu, Jihong Tang, Bin Jiao, Shen Liu

**Affiliations:** ^1^Key Laboratory of Integrated Pest Management of Tropical Crops, Ministry of Agriculture and Rural Affairs, Environment and Plant Protection Institute, Chinese Academy of Tropical Agricultural Sciences, Haikou, China; ^2^Hainan Key Laboratory for Biosafety Monitoring and Molecular Breeding in the Nanfan Area, Sanya Research Institute, Chinese Academy of Tropical Agricultural Sciences, Sanya, China; ^3^State Key Laboratory for Biology of Plant Diseases and Insect Pests, Institute of Plant Protection, Chinese Academy of Agricultural Sciences, Beijing, China; ^4^State Key Laboratory of Ecological Pest Control for Fujian and Taiwan Crops, College of Plant Protection, Fujian Agriculture and Forestry University, Fuzhou, China

**Keywords:** diversity, ecological relationships, high-throughput sequencing, plant virus transmission, thrips vectors, virome

## Abstract

**Introduction:**

Thrips are key vectors for plant viruses, representing a significant challenge to the cultivation of cucurbits and other vegetables in tropical agriculture. This study investigates the diversity of viromes carried by thrips and their ecological roles in viral epidemics affecting specific crops.

**Methods:**

We identify thrips populations in tropical regions and perform a comprehensive virome analysis through high-throughput sequencing.

**Results:**

Our findings reveal that the predominant thrips species associated with these crops are *Frankliniella intonsa*, *Thrips palmi*, and *Megalurothrips usitatus*. The sequencing efforts identified 19 viruses within these thrips, including previously undocumented viruses, such as a double-stranded RNA virus and several positive- and negative-sense single-stranded RNA viruses. Notably, detection rates of specific plant viruses—Melon yellow spot virus (MYSV), Watermelon silver mottle virus (WSMoV), and Telosma mosaic virus (TeMV)—exhibit significant correlations with thrips population density in cucurbits and other vegetables.

**Discussion:**

This study lays the groundwork for future research into the ecological relationships between thrips and plant viruses, offering valuable insights for developing targeted disease management strategies in tropical agricultural systems.

## 1 Introduction

Outbreaks of plant viruses present a serious threat to the production of cucurbits and other vegetables (He and Creasey Krainer, 2020). Viral infections in plants can lead to various symptoms, such as dwarfing, chlorosis, necrosis, yellowing, and mosaic patterns. Infected fruits often exhibit reduced size and surface deformities or mottling, significantly lowering their market value (Ertunç, 2020). Climate change, shifts in planting structures, and evolving farming systems have expanded the cultivation of cucurbits and vegetables, amplifying the impact of viral diseases, which have now become the primary disease challenge for these crops (Jones, 2021). These viruses frequently co-occur in crop fields, resulting in mixed infections that pose a significant risk to production, especially within the Cucurbitaceae, Solanaceae, Fabaceae, Brassicaceae, and Malvaceae families (Sánchez-Sánchez et al., 2024).

Thrips (Thysanoptera) are small, cryptic, piercing-sucking insects known for rapid reproduction and high insecticide resistance, making them prominent agricultural pests worldwide (Mound and Morris, 2007). Importantly, thrips act as vectors for plant viruses, especially in cucurbits and other vegetables, where viral infections they transmit often cause far greater harm than direct feeding damage (Whitfield et al., 2005). Although only a subset of thrips species are virus vectors, they carry an exceptionally diverse range of viruses (Rotenberg et al., 2015). In cucurbits and vegetables, thrips are known to transmit over 15 harmful viruses [including Tobacco streak virus (TSV), Tobacco ringspot virus (TRSV), and Sowbane mosaic virus (SoMV)]. The most impactful of these belong to the Tospovirus genus (now Orthotospovirus, containing 26 recognized species), along with viruses from four additional genera (Nichol et al., 2005). This virus group alone contributes to substantial global economic losses annually (Wu et al., 2018; He et al., 2020). The extensive spread of these viruses underscores thrips’ critical role in viral disease epidemics affecting cucurbits and other vegetables.

Theoretically, vector population size directly impacts transmission efficiency, as larger populations can amplify both the scope and rate of virus spread, assuming the proportion of viruliferous individuals remains relatively stable (Cunniffe et al., 2021). The relationship between thrips density and viral disease prevalence is well-documented (He et al., 2020). Field studies have shown that epidemics of viruses such as Tomato spotted wilt virus (TSWV), Impatiens necrotic spot virus (INSV), and Tomato chlorotic spot virus (TCSV) are closely associated with outbreaks of *Frankliniella intonsa* (Karthikeyan et al., 2021). Similarly, viral disease prevalence in sweet peppers is highly correlated with adult densities of *Thrips palmi* (Zhang et al., 2016). In tropical agriculture, the warm and humid conditions favor rapid disease spread, making viral infections particularly challenging (Sastry and Zitter, 2014). While previous studies highlight the role of thrips in virus transmission, systematic analyses of the viromes they carry and their ecological relationships with viral epidemics in cucurbits and other vegetables are still lacking.

In this study, we investigated tropical vegetable fields on Hainan Island, China’s only tropical island. We first identified and classified thrips populations across these fields in Hainan Island. We then conducted a comprehensive virome analysis to detect and classify viruses within these populations, exploring their phylogenetic relationships. Finally, we analyzed correlations between virus presence in thrips viromes and thrips population densities in cucurbit and vegetable crops. Through these efforts, this study aims to provide foundational insights into the diversity of viral spectra within thrips populations and to elucidate their ecological roles in viral epidemics affecting cucurbits and vegetables.

## 2 Materials and methods

### 2.1 Sampling thrips in cucurbits and other vegetables in Hainan Province, China

Random sampling was conducted in cucurbit and other vegetable fields across Hainan Province, China, a region with a tropical monsoon climate that remains consistently warm and humid year-round. On the island, cucurbits and vegetables hold significant economic importance. Sampling involved selecting five points within each field and examining three plants at each point. Thrips were collected by gently tapping the flowers, leaves, and shoot tips of plants over a white enamel pan, ensuring comprehensive sampling of all accessible plant structures. Dislodged thrips were transferred as a group into a 0.5 L glass jar with a breathable lid using a fine brush. Simultaneously, plant parts hosting thrips were also collected, with records kept of the collection location, host plant species, and relevant field details (Supplementary Table 1). In the laboratory, thrips were examined microscopically for morphological identification (Supplementary Table 2).

For specimens not identifiable by morphology, DNA was extracted using an animal DNA extraction kit (Tiangen Biotech, Beijing, China) for molecular identification. PCR amplification of the COI gene sequence was conducted using universal primers, LepF1 and LepR1, as outlined by Hebert et al. (2004), producing a ~700 bp fragment. Post-amplification, 2 μL of the product was analyzed by gel electrophoresis for verification. The amplified sequences were then compared with those in the NCBI nucleotide database. Thrips were identified to the species level if sequence similarity to reference sequences exceeded 95%. Morphological identification was additionally performed as a complementary approach to molecular methods, further confirming species identity and enhancing the overall accuracy of the results.

### 2.2 Analysis of viromes in thrips

#### 2.2.1 RNA extraction, library construction, and sequencing

Thrips samples were collected from 16 distinct sites on Hainan Island, encompassing various crops, including cucurbits, with sampling conducted between July and December. Total RNA was extracted separately from each thrips population at these sites, with each population containing a minimum of 50 individuals. When multiple thrips species were present at a site, individuals were pooled by species for RNA extraction and analysis. RNA extraction was carried out using the Trizol method, and RNA quantity was assessed with a NanoDrop 2000 spectrophotometer.

To ensure data representativeness and scientific rigor, RNA from the 16 thrips populations was pooled into six composite RNA samples based on thrips species and host plant. This was due to limited RNA, high costs, and our broad research aim. These pooled samples were prepared for metatranscriptomic sequencing. Total RNA samples underwent ribosomal RNA removal using the Ribo-Zero Gold kit (specific for human/mouse/rat rRNA), and paired-end 150 bp sequencing was performed on the Illumina NovaSeq^™^ 6000 platform. Library preparation and sequencing were completed by Lianchuan Biotechnology Co., Ltd. (Hangzhou, China), yielding 81 GB of raw sequencing data. This sequencing depth provides robust data for the identification and comprehensive analysis of thrips virome diversity.

#### 2.2.2 Sequence assembly and discovery of new viruses

After assessing the quality of raw sequencing data from each library, data trimming was performed using Trimmomatic v0.32 (Bolger et al., 2014) to remove low-quality reads and adapter sequences, resulting in a set of clean reads. To efficiently detect viral fragments, these clean reads were initially aligned to the host genome (*F. intonsa* reference genome) using Bowtie2 v2.4.4 (Langmead and Salzberg, 2012), allowing for the removal of host-derived sequences. The remaining, non-aligned reads were then assembled *de novo* using Trinity v2.8.5, which generated preliminary viral contigs.

The assembled contigs were annotated using the blastX algorithm in DIAMOND v3.12 with an *E*-value threshold of 1 × 10^−5^, balancing sensitivity and specificity to ensure reliable viral identification while minimizing false positives. The selected viral contigs were subsequently matched against the non-redundant (NR) protein database for initial virus identification. Open reading frames (ORFs) within the viral contigs were identified using NCBI ORFfinder,^1^ and homologous proteins were identified using HMMER v3.3 against the Pfam protein family database and the RNA-dependent RNA polymerase (RdRP) database of RNA viruses. This approach allowed for further classification and identification of novel viruses based on the latest ICTV species classification standards.

Finally, to estimate viral abundance across the libraries, the quality-controlled paired-end reads were mapped to the identified viral genomes using Bowtie2. Alignments were processed using Samtools v0.1.1 (Li et al., 2009), and RPKM and FPKM values for each contig were calculated using Salmon, providing quantitative insights into viral prevalence within the libraries.

#### 2.2.3 Sanger sequencing

Primers specific to each identified virus were designed for Sanger sequencing. Total RNA from the six libraries obtained in section 2.2.1 was subjected to RT-PCR to synthesize cDNA, with an expected product size of 400 to 600 bp and an annealing temperature (Tm) of 55–60°C. To address potential challenges in primer design, such as complex secondary structures or low copy numbers, four primer pairs were designed for distinct regions of each viral sequence. Each primer pair was tested sequentially to achieve the desired bands; if none of the four pairs yielded a band, the virus was considered absent in that sample. Primer design was conducted using Primer-BLAST,^2^ and primer sequences are provided in Supplementary Table 2. Detailed reaction conditions are as follows: The Premix Taq PCR reaction system consisted of the following components with their respective volumes. Premix Taq was 12.5 μL, cDNA was 1 μL, F-primer was 1 μL, R-primer was 1 μL, and ddH_2_O was added to make the total volume up to 25 μL.

The PCR conditions included an initial denaturation at 95°C for 5 min, followed by 35 cycles of 30 s at 95°C, 40 s at 55°C, and 40 s at 72°C, concluding with a final extension at 72°C for 10 min. PCR products were analyzed using 1.2% agarose gel electrophoresis. If the target band was observed, it was excised from the gel, and the cDNA was purified. Purified products were sent to BGI-Shenzhen for sequencing. Sequencing results were aligned using Geneious v9.0.2, and sequences with similarity above 95% were considered indicative of the virus’s presence.

#### 2.2.4 Phylogenetic tree construction

Phylogenetic analysis was performed by aligning conserved viral sequences identified in this study with known sequences from the same viral families using MAFFT v7.407 with the E-INS-I algorithm. Special focus was placed on RNA-dependent RNA polymerase (RdRp) sequences of RNA viruses, as these are key conserved regions useful for differentiating RNA virus types. Unaligned sequences and poorly aligned regions were removed with TrimAl v1.4 to enhance phylogenetic accuracy. The phylogenetic tree was then constructed using the maximum likelihood (ML) method in IQ-TREE v2.0.3, with bootstrap analysis conducted 1,000 times to validate the reliability of the results. The amino acid substitution model was selected based on the best-fit analysis provided by ModelTest.

#### 2.2.5 Identification of non-viral microorganisms

Non-viral reads were classified and annotated using Kraken v2.0.7-beta 47 (Wood and Salzberg, 2014). For accurate microbial identification, rRNA reads were filtered from the dataset using SortmeRNA (Kopylova et al., 2012). The filtered rRNA reads were then compared to the nucleotide database using the NCBI BLAST suite, applying an E-value threshold of 10^−3^. The microorganism with the lowest *E*-value match was identified as the closest species.

### 2.3 Correlation between the detection of relevant viral diseases in cucurbits and other vegetable crops and the occurrence of thrips

#### 2.3.1 Detection of target viruses in cucurbits and other vegetable crops

Between July 2021 and December 2022, we conducted a survey on the occurrence of viral diseases in cucurbit and other vegetable fields in Hainan, China (Supplementary Table 1). During this period, both thrips and plant samples were collected simultaneously, allowing for a direct comparison of virus presence in thrips populations with the incidence of viral symptoms in corresponding plant samples. A total of 543 leaf samples exhibiting symptoms such as dwarfism, mosaic patterns, malformation, yellow spots, or necrosis were collected and stored at −80°C for further analysis. DNA and RNA were extracted using a plasmid DNA extraction kit (Tiangen Biotech, Beijing, China) and the Trizol method, respectively.

Four target plant viruses (WSMoV, MYSV, WGMMV, TeMV), previously identified in the thrips virome, were detected in these plant tissue samples using reverse transcription and PCR. PCR products were analyzed via agarose gel electrophoresis, with target DNA bands excised and purified for sequencing. Detected plant viruses were then identified through sequence alignment with the NCBI nucleotide database. Detection rates for suspected viral samples were grouped by plant family to evaluate the prevalence of target viruses in cucurbit and other vegetable crops. These four viruses were selected as they were the only plant viruses identified in our initial virome analysis of thrips.

#### 2.3.2 Correlation analysis

We conducted a Pearson correlation analysis to examine the detection of four plant viruses within the *T. palmi* and *F. intonsa* viromes. Specifically, we assessed the correlation between the detection rate of MYSV in cucurbit crops and the occurrence of *T. palmi*. Additionally, we examined the correlation between the detection rate of WSMoV in both Cucurbitaceae and Fabaceae and the abundance of *T. palmi*. In both analyses, virus detection in plant samples was based on presence/absence rather than incidence rates. Similarly, we analyzed the correlation between the detection rate of WGMMV in cucurbit crops and the occurrence of *F. intonsa*, as well as the correlation between the detection rate of TeMV in leguminous crops and the occurrence of *F. intonsa*.

## 3 Results

### 3.1 Occurrence of thrips in the study region

**Figure 1:**
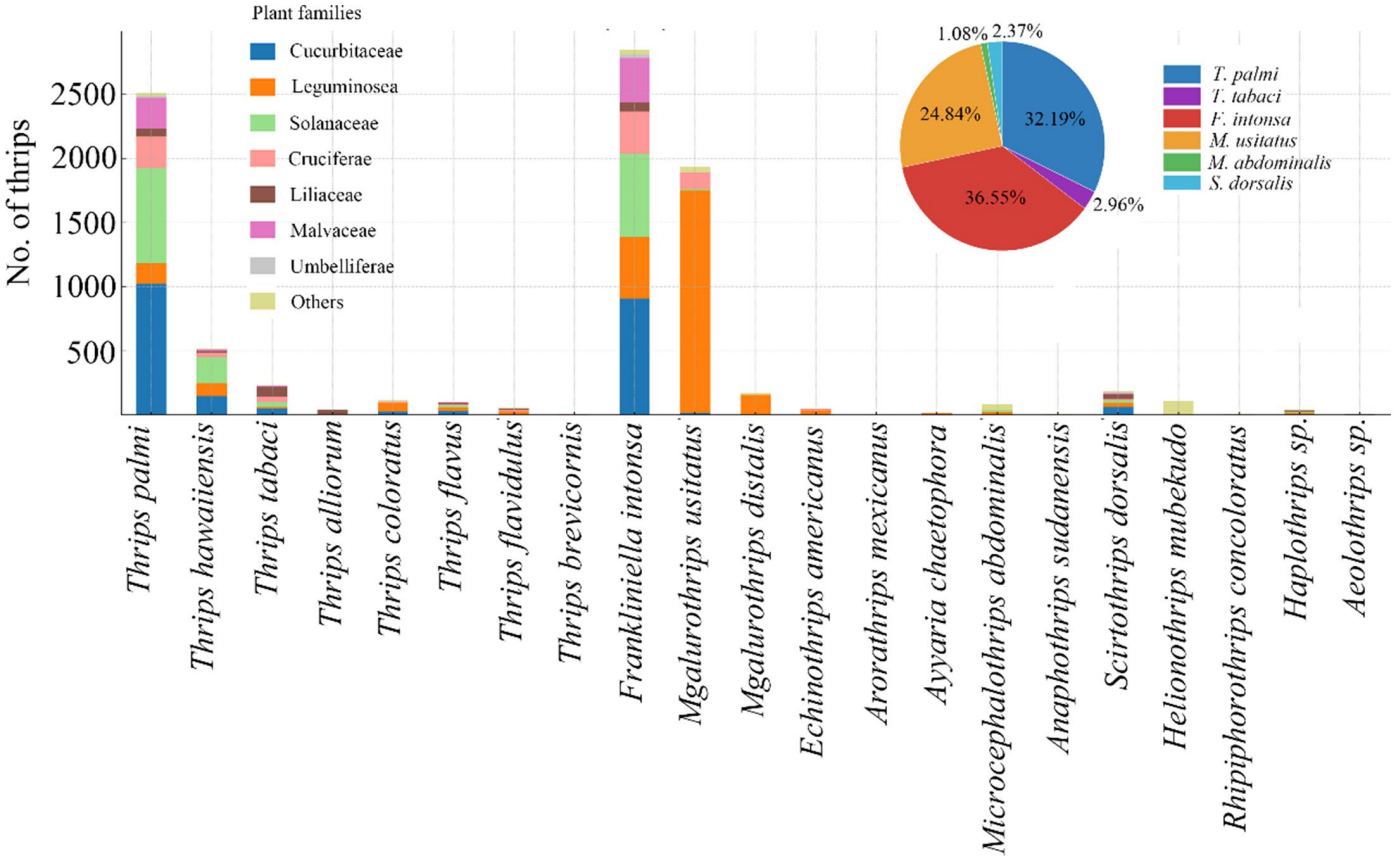
Major thrips species collected in survey on Hainan Island, southern China (pie chart) and their distribution on cucurbits and other vegetables (by family, see bar chart).

Thrips were observed on crops across seven plant families: Cucurbitaceae, Fabaceae, Solanaceae, Brassicaceae, Liliaceae, Malvaceae, and Apiaceae, with 10, 14, 9, 12, 12, 6, and 5 thrips species identified in each, respectively. Additionally, 15 thrips species were identified on other crops. Among the thrips collected throughout the survey, *F. intonsa*, *T. palmi*, and *M. usitatus* were the dominant species, accounting for 36.55, 32.19, and 24.84% of the total, respectively. *F. intonsa* and *T. palmi* primarily infested cucurbit crops, whereas *M. usitatus* was most prevalent on leguminous crops (Figure 1).

### 3.2 Analysis of thrips viromes

#### 3.2.1 Virus identification

From each of the six thrips sample libraries, a total of 12.88 to 13.80 GB of data were generated, reflecting the data range across different species. After filtering out low-quality reads, 65,532,884 clean reads were retained. Through *de novo* assembly, reference genome alignment, and BLASTx searches, we identified 36,269 initial viral contigs, with lengths ranging from 200 to 20,390 nt and an average length of 502 nt. Following iterative extension using Bowtie2 and Samtools, and after rigorous screening and quality control, we obtained 30 high-quality viral sequences from the six sample pools, identifying 19 distinct viruses. These viruses were categorized into nine viral families, with three unclassified viral groups comprising one double-stranded RNA virus (*Totiviridae*), 11 positive-sense single-stranded RNA viruses, six negative-sense single-stranded RNA viruses, and one unclassified RNA virus (Supplementary Table 3). Among the identified viruses, negative-sense single-stranded RNA viruses were the most prevalent. *Picornavirales* accounted for 35.63% of total viral reads, followed by *Amarillovirales* (27.80%) and *Martellivirales* (16.06%) (Table 1).

**Table 1:** Relative abundance of viral classifications in libraries.

Nucleic acid type	Order	Reads	Relative abundance (%)
dsRNA viruses	*Ghabrivirales*	8,504	5.28
−ssRNA viruses	*Patatavirales*	15,488	9.62
	*Amarillovirales*	44,768	27.80
	*Hepelivirales*	1,570	0.97
	*Picornavirales*	57,369	35.63
	*Martellivirales*	25,858	16.06
+ssRNA viruses	*Mononegavirales*	3,666	2.28
	*Bunyavirales*	3,162	1.96
Unclassified RNA viruses	unclassified	650	0.40

#### 3.2.2 Viral community composition

**Figure 2:**
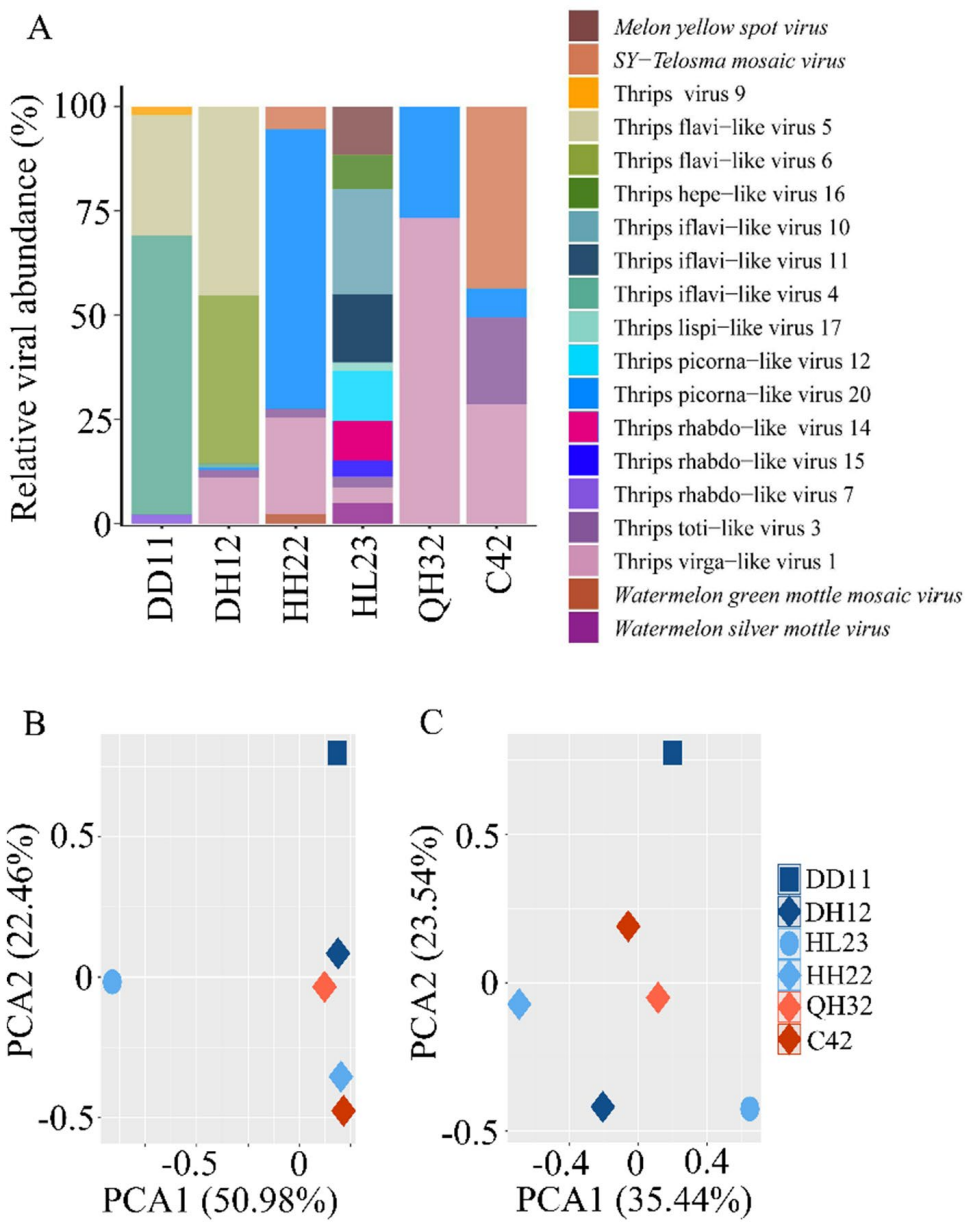
Comparison of viral and bacterial communities across different thrips samples. **(A)** Comparison of viral species and abundance across different thrips sample libraries. Principal component analysis (PCA) of the viral **(B)** and bacterial **(C)** communities in the different thrips samples.

Based on host categories, the viruses carried by thrips were classified as fungal viruses (one species), plant viruses (four species), arthropod viruses (13 species), and vertebrate viruses (one species) (Supplementary Table 3). The four plant viruses identified were MYSV, WSMoV, TeMV, and WGMMV, with MYSV and WSMoV detected in *T. palmi*, and TeMV and WGMMV in *F. intonsa* (Figure 2A).

Analysis of the viral community composition in the three thrips samples revealed significant differences in virus species and abundance among samples (*p* < 0.05). In the *T. palmi* sample (HL23), 11 viruses were detected, with *Thrips iflavi-like virus 10* being the most abundant, accounting for 25.26% of the total. In the *F. intonsa* samples (DH12, HH22, QH32, C42), between 2 and 6 viruses were detected, each dominated by a different virus: *Thrips flavilike viruses 5* and *6* in DH12, *Thrips picorna-like virus 20* in HH22, *Thrips virga-like virus 1* in QH32, and *SY-Telosma mosaic virus* in C42. In the *M. usitatus* sample (DD11), four viruses were identified, with *Thrips iflavi-like virus 4* being the most prevalent, comprising 66.84% of the total (Figure 2A).

Principal component analysis (PCA) demonstrated that viral community compositions differed significantly among the three thrips species (*p* < 0.05), while viral composition was similar among thrips on the same host plant family. This finding suggests that viral communities are primarily influenced by the host plant (Figures 2B,C).

#### 3.2.3 Identification and analysis of dsRNA viruses

**Figure 3:**
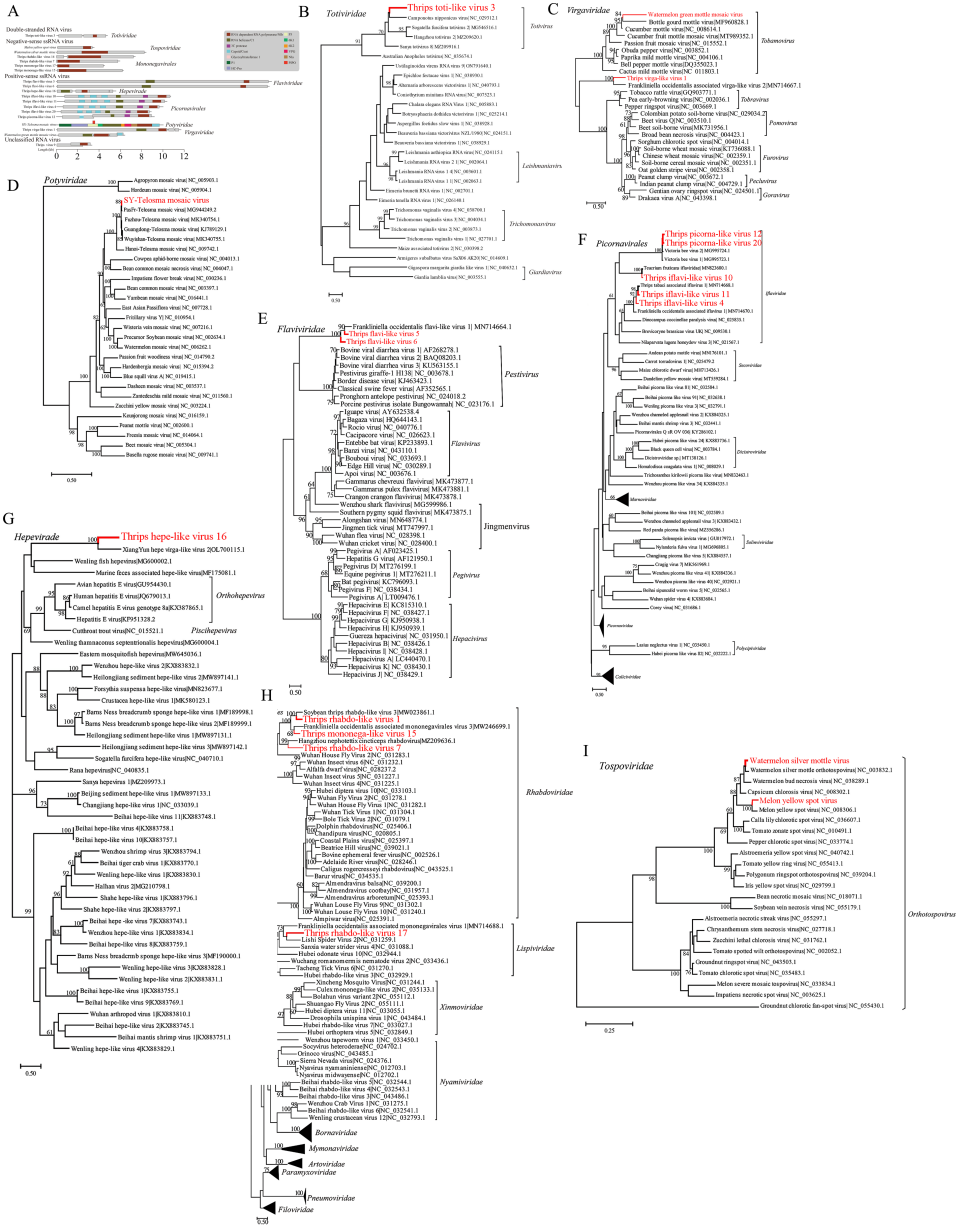
Presentation of the genomic structure of double-stranded RNA viruses, and the phylogenetic relationships of viruses from multiple families (including *Virgaviridae*, etc.), negative-sense single-stranded RNA viruses, and *Tospoviridae* viruses identified in this study with known counterparts. Schematic of viral genome structure and phylogenetic relationships of double-stranded RNA viruses **(A,B)**. **(A)** Schematic representation of the viral genome structure. **(B)** Phylogenetic relationships of the double-stranded RNA viruses identified in this study (marked in red) with other known viruses in the *Totiviridae* family. Phylogenetic relationships of viruses identified in this study with known viruses across multiple families **(C–G)**. **(C)** Phylogenetic relationships of the *Virgaviridae* viruses identified in this study (marked in red) with other known viruses in the *Virgaviridae* family. **(D)** Phylogenetic relationships of the *Potyviridae* viruses identified in this study (marked in red) with other known viruses in the *Potyviridae* family. **(E)** Phylogenetic relationships of the *Flaviviridae* viruses identified in this study (marked in red) with other known viruses in the *Flaviviridae* family. **(F)** Phylogenetic relationships of the *Picornavirales* viruses identified in this study (marked in red) with other known viruses in the *Picornavirales* order. **(G)** Phylogenetic relationships of the *Hepeviridae* viruses identified in this study (marked in red) with other known viruses in the *Hepeviridae* family. Phylogenetic relationships of negative-sense single-stranded RNA viruses and *Tospoviridae* viruses identified in this study **(H,I)**. **(H)** Phylogenetic relationships of the negative-sense single-stranded RNA viruses identified in this study (marked in red) with other known viruses in the *Mononegavirales* order. **(I)** Phylogenetic relationships of the *Tospoviridae* viruses identified in this study (marked in red) with other known viruses in the *Tospoviridae* family.

In three *F. intonsa* sample libraries (C42, DH12, HH22) and one *T. palmi* sample library (HL23), we identified a novel double-stranded RNA (dsRNA) virus in the *Totiviridae* family, tentatively named *Thrips toti-like virus 3* (TTV3). The full genome of TTV3 is 4,858 nucleotides long and contains two open reading frames (ORFs). This specific ORF arrangement in the TTV3 genome is significant as it provides the basis for further exploring the virus’s replication mechanism and its interaction with the host thrips. The first ORF encodes a protein of unknown function with a molecular mass of 81.072 kDa, while the second ORF encodes an RNA-dependent RNA polymerase (RdRp) (Figure 3A). Phylogenetic analysis shows that TTV3 forms a distinct evolutionary branch within the *Totivirus* genus, exhibiting 36.83% amino acid sequence similarity to *Camponotus nipponicus virus*, a virus found in tree-dwelling ants (Figure 3B).

#### 3.2.4 Identification and analysis of +ssRNA viruses

Twelve positive-sense single-stranded RNA (+ssRNA) viruses were identified, representing various families, including *Virgaviridae*, *Potyviridae*, *Flaviviridae*, *Picornavirales*, and *Hepeviridae*. In one *F. intonsa* sample (HH22), a sequence closely matching *Watermelon green mottle mosaic virus* (WGMMV) was detected, with 99% amino acid homology (Figure 3C). Additionally, a novel virus related to *Virgaviridae*, identified in multiple thrips samples (DH12, HH22, QH32, C42, HL23), was named *Thrips virga-like virus 1* (TVV1). The TVV1 genome is approximately 11,938 nucleotides in length and contains three open reading frames (ORFs), one of which includes both Hel and RdRp domains. TVV1 shares 42.8% amino acid similarity with *F. intonsa*-associated virga-like virus 2 (FAVV2) and forms a distinct evolutionary branch with FAVV2 (Figure 3C).

In two *F. intonsa* samples (C42 and HH22), sequences of *Telopea mosaic virus* (TeMV) from the *Potyvirus* genus were identified and designated SY-TeMV. The SY-TeMV genome is 10,057 nucleotides long, with a 9,630-nucleotide ORF encoding 3,209 amino acids. Phylogenetic analysis reveals that SY-TeMV forms a monophyletic branch with other Chinese isolates (Figure 3D).

Furthermore, two novel *Flavivirus* sequences were isolated from *F. intonsa* and *M. usitatus* samples, designated *Thrips flavi-like virus 5* (TFV5) and *Thrips flavi-like virus 6* (TFV6), respectively. The genomes of these viruses are notably longer than those of other members in the *Flaviviridae* family (Figure 3C). Phylogenetic analysis suggests that TFV5 and TFV6 are distantly related to other insect-specific flaviviruses (Figure 3E).

In the *Picornavirales* order, *Thrips iflavi-like virus 4* (TIV4) and *Thrips picorna-like virus 20* (TPV20) were detected in *M. usitatus* and *F. intonsa* samples, respectively. Additionally, four novel viruses were isolated from the *T. palmi* sample, including *Thrips iflavi-like virus 10* (TIV10), *Thrips iflavi-like virus 11* (TIV11), and *Thrips picorna-like virus 12* (TPV12). These viruses belong to the *Iflaviridae* family but occupy distinct evolutionary branches (Figure 3F).

Lastly, a novel *Hepeviridae* virus, named *Thrips hepe-like virus 16* (THV16), was identified in the *T. palmi* sample. The THV16 genome is 5,802 nucleotides long and contains three ORFs. Phylogenetic analysis indicates that THV16 is distantly related to other hepatitis viruses (Figure 3G).

#### 3.2.5 Identification and analysis of −ssRNA viruses

Four novel virus sequences, suspected to belong to the negative-sense single-stranded RNA (−ssRNA) viruses, were identified. Among these, one virus was detected in a *T. palmi* sample (HL23) and was named *Thrips mononega-like virus 17* (TMV17), potentially a new member of the *Lispiviridae* family. The other three viruses were detected in *M. usitatus* (DD11) and *T. palmi* (HL23) samples, showing amino acid sequence homology with viruses from the *Rhabdoviridae* family, ranging from 42.36 to 52.62%. These viruses were named *Thrips rhabdo-like virus 7* (TRV7), *Thrips rhabdo-like virus 14* (TRV14), and *Thrips mononega-like virus 15* (TMV15), respectively. Due to limited sequencing depth, their full genome structures remain unresolved, and they were identified as vOTUs (virus operational taxonomic units) based solely on the conserved sequence of RNA-dependent RNA polymerase (RdRp).

Phylogenetic analysis revealed that TMV17 is most closely related to *Lishi Spider Virus 2* and *F. intonsa associated mononegavirales virus 1*, with nucleotide and amino acid sequence similarities of 29.71 and 29.73%, respectively. TRV7, TRV14, and TMV15 clustered with other arthropod-hosted *Rhabdoviridae* members, distinct from viruses infecting vertebrate and plant hosts. Notably, TRV14 is most closely related to *Soybean Thrips rhabdo-like virus 3*, which infects soybean thrips, with 49.42% amino acid similarity. TMV15 shows 41.12% amino acid similarity to *F. intonsa associated mononegavirales virus 3*, which infects *F. intonsa*, suggesting that these thrips-associated viruses may represent a new evolutionary branch (Figure 3H).

Additionally, two full-length sequences with high similarity to the LRNA segment of *Orthotospovirus* were obtained from the *T. palmi* sample (HL23). These sequences were most closely related to *WSMoV* and *MYSV*, with nucleotide and amino acid sequence similarities of 95.06 and 96.42%, respectively (Figure 3I).

### 3.3 Detection of target viruses in cucurbits and other vegetables and their correlation with thrips occurrence

**Figure 4:**
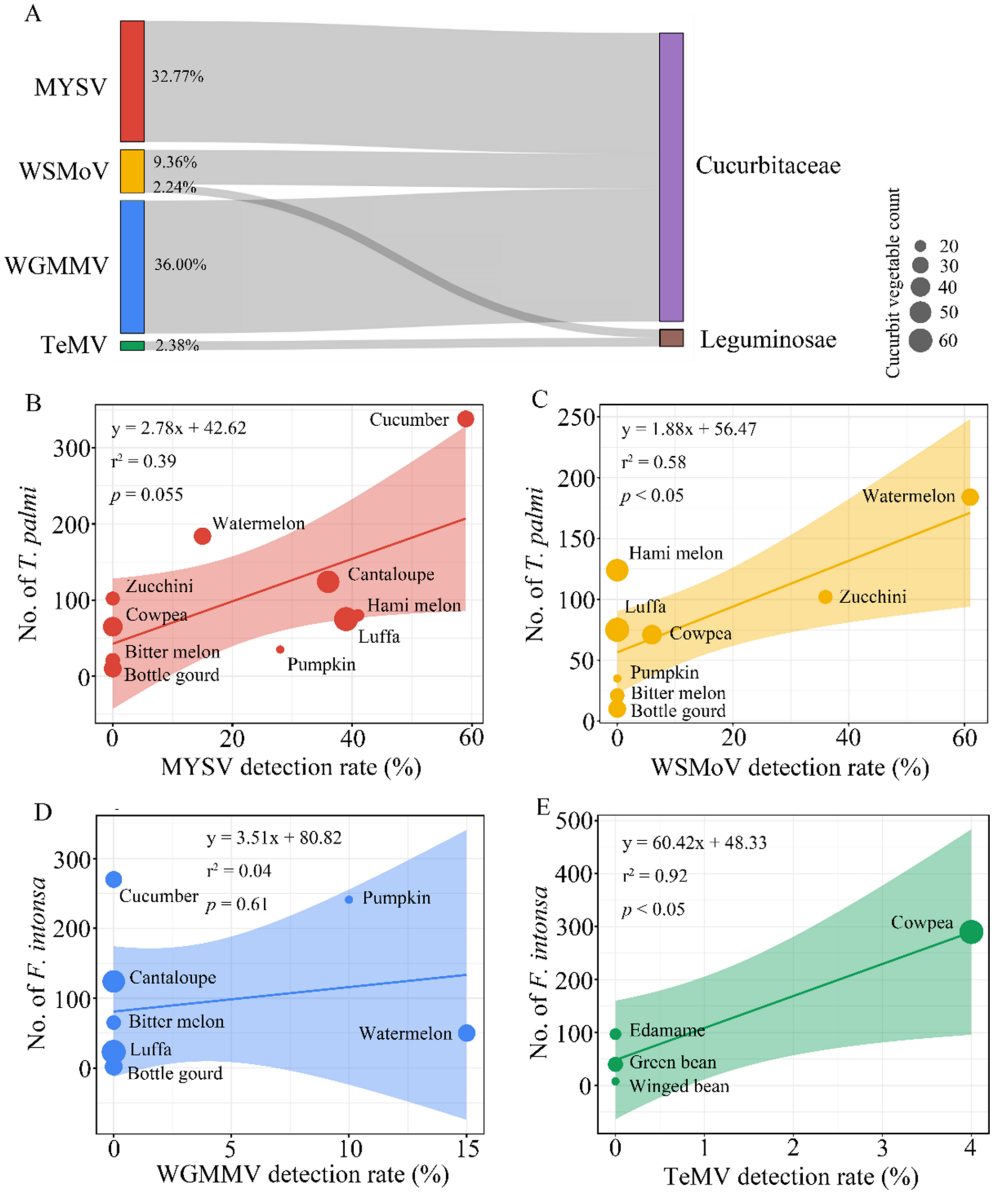
Correlation analysis between thrips-borne plant viruses and different families of cucurbit and leguminous crops. **(A)** Distribution of four plant viruses in cucurbit and leguminous crops. **(B–E)** Correlation between the detection rates of four plant viruses in cucurbit and leguminous crops and the occurrence of thrips on these crops. The size of the scatter points represents the number of samples tested, and the shaded areas indicate the 95% confidence intervals. **(B)** Correlation between the detection rate of MYSV and the abundance of *T. palmi* on cucurbit crops. **(C)** Correlation between the detection rate of WSMoV and the abundance of *T. palmi* on cucurbit and leguminous crops. **(D)** Correlation between the detection rate of WGMMV and the abundance of *F. intonsa* on cucurbit crops. **(E)** Correlation between the detection rate of TeMV and the abundance of *F. intonsa* on leguminous crops.

*MYSV*, *WSMoV*, and *WGMMV* viruses were detected exclusively in cucurbit crops, with detection rates of 32.77, 9.36, and 36.00%, respectively, while *WSMoV* and *TeMV* were detected solely in leguminous crops, with detection rates of 2.24 and 2.38%, respectively (Figure 4A). These findings suggest that these viruses may exhibit specific host preferences. The detection rate of MYSV showed a weak positive correlation with the presence of *T. palmi* (*r*^2^ = 0.39, *p* = 0.055), with a detection rate of 59% in cucumber (Figure 4B). In contrast, the detection rate of *WSMoV* was significantly and positively correlated with *T. palmi* occurrence (*r*^2^ = 0.58, *p* < 0.05), with the highest detection rate in watermelon at 61%, and a 6% detection rate in cowpea (Figure 4C). The detection rate of *WGMMV* did not show a significant correlation with the presence of *F. intonsa* (*r*^2^ = 0.04, *p* = 0.61) (Figure 4D). However, *TeMV* displayed a significant positive correlation with *F. intonsa* occurrence (*r*^2^ = 0.92, *p* < 0.05), with a 4% detection rate in cowpea (Figure 4E). These results suggest that these viruses may exhibit host specificity in particular crops and that their prevalence appears to be correlated with the occurrence of specific thrips populations.

## 4 Discussion

In this study, we investigated the occurrence of thrips on crops from seven plant families in the tropical region of Hainan, focusing on the three dominant thrips species: *F. intonsa*, *M. usitatus*, and *T. palmi*. Virome analysis of six thrips sample libraries revealed 19 viruses spanning nine viral families and three unclassified viral groups, including double-stranded RNA viruses, positive-sense single-stranded RNA viruses, and negative-sense single-stranded RNA viruses. Our study also identified several novel viruses, including *Ghabrivirales* (*Totiviridae*), a double-stranded RNA virus and multiple positive- and negative-sense single-stranded RNA viruses. Additionally, detection rates of four plant viruses (MYSV, WSMoV, WGMMV, and TeMV) were significantly correlated with thrips abundance, suggesting a potential association between thrips density and the epidemiology of viral diseases in cucurbits and other vegetables.

We identified a total of 19 viruses in the thrips virome, spanning plant, arthropod, fungal, and vertebrate viruses. This finding aligns with previous studies in other arthropods, such as bees, mosquitoes, and locusts, further supporting the role of invertebrates, particularly arthropods, as significant hosts for RNA viruses (Chiapello et al., 2021; Thekke-Veetil et al., 2020; Xu et al., 2022). Additionally, we discovered several novel viruses, including two new members of the *Flaviviridae* family, TFV5 and TFV6, which form distinct evolutionary branches in the phylogenetic tree and may represent new viral lineages within Thysanoptera (Tree et al., 2016). These novel findings underscore the complexity of thrips as virus vectors and highlight their potential impacts on ecosystems.

The significant differences in viral read abundance across thrips samples may stem from varying infection rates at different collection stages, as well as the possibility that some low-abundance viruses are not actively replicating within thrips but are passively acquired from feeding on virus-contaminated plants (Neri et al., 2022). The high detection rate of abundant viruses, such as *Picornavirales*, suggests these viruses likely complete their life cycle within thrips, indicating active replication (Neri et al., 2022). Conversely, the low abundance of certain viruses may result from their integration into the thrips genome as endogenous viral elements, a phenomenon also observed in other arthropods (Neri et al., 2022). The discovery of these new viral species not only broadens our understanding of the diversity within the thrips virome but also underscores the complex interactions between thrips and the viruses they harbor. These findings offer valuable insights for future research on viral evolution, particularly in understanding virus adaptation to insect hosts and its potential implications for virus transmission dynamics in agricultural ecosystems.

The study results indicate that the composition of thrips viral communities is primarily influenced by their host plants, with viral diversity in different thrips populations closely linked to host plant diversity (Thongsripong et al., 2021). This finding supports the theory of virus-host co-evolution, suggesting that viruses establish specific ecological relationships with their hosts through long-term evolutionary processes (Brooks et al., 2017). The greater diversity and abundance of viruses carried by *T. palmi* compared to *F. intonsa* and *M. usitatus* may be attributed to *T. palmi*’s broader range of host plants, providing increased exposure to diverse viral species. Additionally, *T. palmi* may possess biological or ecological traits—such as a longer lifespan, higher mobility, or more frequent feeding on varied plant species—that enhance its efficiency as a virus vector, thus increasing its likelihood of acquiring and transmitting a broader array of viruses. These findings hold significant implications for pest management. The higher viral diversity associated with *T. palmi* suggests that this species should be a primary focus for monitoring and control efforts, particularly in crops where it is prevalent. Targeting *T. palmi* populations could help reduce the transmission of multiple plant viruses, thereby lowering the overall viral burden in agricultural ecosystems.

Using high-throughput sequencing, we detected four plant viruses—MYSV, WSMoV, WGMMV, and TeMV—in *T. palmi* and *F. intonsa* samples. Previous studies have demonstrated that MYSV and WSMoV are effectively transmitted by *T. palmi*, confirming the natural transmission of these viruses (Mou et al., 2021). However, the transmission mechanisms of WGMMV and TeMV remain unclear. While studies suggest that a close relative of WGMMV, CGMMV, is seed-transmitted (Yang et al., 2018), no definitive transmission pathway has been reported for WGMMV or TeMV. This study is the first to detect WGMMV and TeMV in *F. intonsa* samples, suggesting that *F. intonsa* may have come into contact with these emerging viruses. However, detecting these viruses in thrips does not necessarily imply that thrips can transmit them. Thrips may acquire viral particles by feeding on infected plants, but this does not confirm their ability to transmit the viruses to new hosts. Further research is needed to explore the specific transmission mechanisms and efficiency of these viruses, including whether *F. intonsa* can actively transmit WGMMV and TeMV, to better understand their transmission dynamics in the field.

Insects, particularly thrips, are significant vectors of plant viruses, with strong transmission capabilities and widespread distribution, profoundly impacting the field epidemiology and spread of viruses (Jones, 2005). This study demonstrates that the field prevalence of MYSV is significantly and positively correlated with the population size of *T. palmi*, suggesting that *T. palmi* may contribute to the transmission of MYSV in cucurbits and other vegetables. However, virus transmission is influenced by various factors, including virus acquisition efficiency, environmental conditions, and the presence of additional vectors. Further research is necessary to clarify the role of *T. palmi* in this process. Similarly, the correlation between WSMoV spread and *T. palmi* is weaker, potentially due to factors such as initial infection sources, host plant resistance, and other ecological variables. Additionally, the detection of WGMMV and TeMV in *F. intonsa* samples suggests that *F. intonsa* may be involved in their transmission, but further studies are required to elucidate the specific mechanisms.

This study enhances our understanding of the diverse range of viruses carried by thrips and reveals significant correlations between thrips populations and virus prevalence in cucurbits and other vegetables. While these correlations do not confirm causation, they offer valuable insights into the potential ecological interactions between thrips and plant viruses. These findings provide a foundation for developing targeted pest management strategies focused on key thrips species and the conditions that influence viral transmission. Future research should seek to elucidate the specific transmission mechanisms and ecological dynamics of these viruses, which will be essential for improving virus control in agricultural ecosystems.

## Data Availability

All raw sequence data generated in this study have been deposited in the NCBI Sequence Read Archive (SRA) under BioProject accession number PRJNA1242210. This BioProject includes six SRA datasets related to thrips collected in the tropical region of Hainan Island, southern China. The data are publicly available and can be accessed at the following link: https://www.ncbi.nlm.nih.gov/bioproject/PRJNA1242210.

## Footnotes

^1^ https://www.ncbi.nlm.nih.gov/orffinder/

^2^ https://www.ncbi.nlm.nih.gov/tools/primer-blast/
