# Supplementary material for: Thrips and plant viruses: a comprehensive virome analysis in tropical agriculture
Source: Front Microbiol. 2025 Apr 28;16:1540883. doi: 10.3389/fmicb.2025.1540883 (PMC12066466; doi:10.3389/fmicb.2025.1540883)
Supplement: Supplementary file 1 [file Table_1.docx]

Supplementary Table 1 Thrips and crop sampling information

| Family | Host plant | Number of thrips | | | | Total |
| --- | --- | --- | --- | --- | --- | --- |
|  |  | Sanya | Ledong | Lingshui | Dongfang |  |
| Leguminosae | *Vigna unguiculata* (L.) Walp*.* | 1084 | 205 | 437 | 57 | 1783 |
|  | *Psophocarpus tetragonolobus* (L.) DC. | 0 | 0 | 25 | 0 | 25 |
|  | *Phaseolus vulgaris* L. | 206 | 43 | 79 | 20 | 348 |
|  | *Glycine max* (L.) Merr | 465 | 251 | 0 | 0 | 716 |
| Cucurbitaceae | *Cucumis sativus* L. | 564 | 137 | 119 | 76 | 896 |
|  | *Citrullus lanatus* (Thunb.) Matsum & Nakai | 103 | 0 | 170 | 0 | 273 |
|  | *Cucurbita moschata* (Duchesne) Duchesne *ex* Poir. | 72 | 123 | 94 | 16 | 305 |
|  | *Cucumis melo* var. saccharinus | 28 | 101 | 0 | 0 | 129 |
|  | *Lagenaria siceraria* (Molina) Standl. | 0 | 0 | 19 | 0 | 19 |
|  | *Luffa cylindrica* M.Roem. | 93 | 0 | 23 | 0 | 116 |
|  | *Momordica charantia* L. | 67 | 26 | 0 | 8 | 101 |
|  | *Cucumis melo* L. | 82 | 146 | 55 | 0 | 283 |
|  | *Cucurbita pepo* L. | 107 | 0 | 34 | 0 | 141 |
| Solanaceae | *Capsicum annuum* L. | 324 | 559 | 101 | 61 | 1045 |
|  | *Solanum melongena* L. | 301 | 67 | 110 | 28 | 506 |
|  | *Solanum lycopersicum* L. | 43 | 10 | 96 | 0 | 149 |
| Liliaceae | *Allium sativum* L. | 69 | 0 | 32 | 0 | 101 |
|  | *Allium ascalonicum* L. | 73 | 24 | 128 | 0 | 225 |
| Cruciferae | *Brassica chinensis* L. | 114 | 46 | 89 | 0 | 249 |
|  | *Rophanus satims* L. | 134 | 315 | 84 | 27 | 560 |
|  | *Brassica chinensis* L. | 10 | 5 | 24 | 0 | 39 |
| Malvaceae | *Abelmoschus esculentus* (L.) Moench | 368 | 242 | 20 | 0 | 630 |
| Umbelliferae | *Apium graveolens* L. | 39 | 0 | 0 | 0 | 39 |
|  | *Coriandrum sativum* L. | 10 | 0 | 0 | 0 | 10 |
| Others | *Zea mayz* L. | 104 | 0 | 0 | 0 | 104 |
|  | *Dioscorea esculenta* (Lour.) Burkill | 56 | 0 | 145 | 0 | 201 |
|  | *Lactuca sativa* L. | 22 | 0 | 6 | 0 | 28 |
|  | Total | 4538 | 2300 | 1890 | 293 | 9021 |

Note: These locations - Sanya, Ledong, Lingshui, and Dongfang - are all South China breeding bases in Hainan Island. They share a warm and tropical climate, , which is ideal for year - round breeding activities, providing crucial settings for agricultural research and variety improvement.

Supplementary Table 2 Verification information table of Sanger sequencing primers

| Virus name | Abbreviation | Sequence (5′-3′) | Product size (bp) |
| --- | --- | --- | --- |
| *Thrips virga-like virus 1 | TVV1 | Forward: TTGTGCAACCAGTTCCCACT  Reverse: CACAACCGTGTTGTTGCGAT | 481 |
| *SY-*Telosma mosaic virus* | SY-TeMV | Forward: TTGGCGTTTCTTTCCTTGCG  Reverse: AATACCTCGCACAGTGGCAA | 457 |
| *Thrips toti-like virus 3 | TTV3 | Forward: CGTGGGGCGCTAGCTATTTA  Reverse: CTCGTCAGCATGGGGAAAGT | 480 |
| *Thrips iflavi-like virus 4 | TIV4 | Forward: GCTGTTTGGAATAGCGAGCG  Reverse: TGGCAGCGCTTGAGGATAAA | 484 |
| *Thrips flavi-like virus 5 | TFV5 | Forward: CGACAACGCTGTTGAAGCAA  Reverse: GGCGTATGTCACCCGATCAT | 488 |
| *Thrips flavi-like virus 6 | TFV6 | Forward: GCAAACTGATAGCGAGCGTG  Reverse: CGATCTGCATGCCAACGAAG | 585 |
| Thrips rhabdo-like virus 7 | TRV7 | Forward: GCAGCGGCCACTAAAATGAG  Reverse: TCATAACCATCAGCCGGAGC | 535 |
| Thrips virus 9 | TV9 | Forward: CCGACAGGGTTCGAGATACG  Reverse: GACCTACGATGATGGCGGAG | 506 |
| *Thrips iflavi-like virus 10 | TIV10 | Forward: CTACGGACCACCACTGGAAC  Reverse: TTCTCTGGGCATTTCCGCAT | 484 |
| *Thrips iflavi-like virus 11 | TIV11 | Forward: AACGACTGCTGTTACTGGGG  Reverse: AGAAGCTGCGTCTTCAACGA | 533 |
| Thrips picorna-like virus 12 | TPV12 | Forward: ATAACGGGTGCCGATTTGGT  Reverse: TAGGAGCCGTTCCGTTTTCC | 459 |
| **Watermelon silver mottle virus* | WSMoV | Forward: AGGAGCTGATCATTTCCGGC  Reverse: GCACCCAGGGTATCCCTTTT | 526 |
| *Thrips rhabdo-like virus 14 | TRV14 | Forward: AGTCTTCCCACAATGGCTCG  Reverse: TGCTTTCAGGGTAGCTGACG | 579 |
| *Thrips mononega-like virus 17 | TMV17 | Forward: CACATCATGCAGGCATTCGG  Reverse: AGGCCTTAGGGTCATGCAAC | 490 |
| Thrips hepe-like virus 16 | THV16 | Forward: ACCACCACACAACTTCGGTT  Reverse: ATTTGTCAGACTCCTGGGCG | 583 |
| *Thrips mononega-like virus 15 | TMV15 | Forward: CCCAGGTGGTGAAGTTTGGT  Reverse: TCCCAGCTAGCTCACACTCT | 529 |
| **Melon yellow spot virus* | MYSV | Forward: ATGCGCCTTTTAATCCACCC  Reverse: CTCTTGAGCCACCTGCAAAC | 510 |
| **Watermelon green mottle mosaic virus* | WGMMV | Forward: GTATCCGGCCACGAATAGCA  Reverse: GCGCGTAAAGCATCAACGAT | 507 |
| *Thrips picorna-like virus 20 | TPV20 | Forward: CTAAGCCGCTGCGTTGAATC  Reverse: ACCGATTTCATTGGCAGGGT | 555 |

*After verification, it really exists.

Supplementary Table 3 Virus information

| Nucleic acid | Order | Family | Virus name | Host |
| --- | --- | --- | --- | --- |
| dsRNA | *Ghabrivirales* | *Totiviridae* | Thrips toti-like virus 3 | Fungus |
| -ssRNA | *Patatavirales* | *Potyviridae* | SY-*Telosma mosaic virus* | Plant |
|  | *Amarillovirales* | *Flaviviridae* | Thrips flavi-like virus 5 | Arthropoda |
|  |  |  | Thrips flavi-like virus 6 | Arthropoda |
|  | *Hepelivirales* | *Hepeviridae* | Thrips hepe-like virus 16 | Vertebrate |
|  | *Picornavirales* | *Iflaviridae* | Thrips iflavi-like virus 4 | Arthropoda |
|  |  |  | Thrips iflavi-like virus 10 | Arthropoda |
|  |  |  | Thrips iflavi-like virus 11 | Arthropoda |
|  |  | Unclassified | Thrips picorna-like virus 12 | Arthropoda |
|  |  |  | Thrips picorna-like virus 20 | Arthropoda |
|  | *Martellivirales* | *Virgaviridae* | *Watermelon green mottle mosaic virus* | Plant |
|  |  |  | Thrips virga-like virus 1 | Arthropoda |
| +ssRNA | *Mononegavirales* | *Lispiviridae* | Thrips lispi-like virus 17 | Arthropoda |
|  |  | *Rhabdoviridae* | Thrips rhabdo-like virus 15 | Arthropoda |
|  |  |  | Thrips rhabdo-like virus 7 | Arthropoda |
|  |  |  | Thrips rhabdo-like virus 14 | Arthropoda |
|  | *Bunyavirales* | *Tospoviridae* | *Watermelon silver mottle virus* | Plant |
|  |  |  | *Melon yellow spot virus* | Plant |
| unknown | unclassified | unclassified | Thrips virus 9 | Arthropoda |
